# Does inflammation moderate the effect of vitamin D supplementation on depressive symptoms? Results of an interventional study in children and adolescents

**DOI:** 10.1007/s00394-026-03920-0

**Published:** 2026-03-31

**Authors:** Laura Schlarbaum, Nicole Jankovic, Judith Bühlmeier, Eva Hohoff, Rhea Dankers, Harald Engler, Raphael Hirtz, Denise Zwanziger, Corinna Grasemann, Triinu Peters, Anke Hinney, Jochen Antel, Manuel Föcker, Lars Libuda

**Affiliations:** 1 Institute of Nutrition, Consumption and Health, Faculty of Natural Sciences, Paderborn University, Warburger Strasse 100, 33098 Paderborn, Germany; 2 Institute for Integrative Health Care and Health Promotion, Faculty of Health, Witten/Herdecke University, Witten, Germany; 3 Institute of Medical Psychology and Behavioral Immunobiology, Center for Translational Neuro- and Behavioral Sciences, University Hospital Essen, University of Duisburg-Essen, Hufelandstrasse 55, 45122 Essen, Germany; 4 Center for Child and Adolescent Medicine, Helios University Hospital Wuppertal, Witten/Herdecke University, Wuppertal, Germany; 5 Division of Pediatric Endocrinology and Diabetology, Department of Pediatrics II, University Hospital Essen, University of Duisburg-Essen, Hufelandstrasse 55, 40211 Essen, Germany; 6 Department of Pediatrics, St‑Josef Hospital Bochum and Center for Rare Diseases, Ruhr‑University Bochum, Alexandrinenstrasse 5, 44791 Bochum, Germany; 7 Central Laboratory, University Hospital Essen (AöR), University of Duisburg-Essen, Hufelandstrasse 55, 45147 Essen, Germany; 8 Department of Pediatrics, Medical Center of the Johannes Gutenberg University Mainz, Mainz, Germany; 9 Section of Molecular Genetics of Mental Disorders, University Hospital Essen, Essen, Germany; 10 Center for Translational Neuro- and Behavioral Sciences, University Hospital Essen, Essen, Germany; 11 Institute of Sex- and Gender-Sensitive Medicine, University Hospital Essen, Essen, Germany; 12 Department of Child and Adolescent Psychiatry, Psychotherapy and Psychosomatics, University Hospital Essen (AöR), University of Duisburg-Essen, Essen, Germany; 13 Department of Child and Adolescent Psychiatry, University Hospital Münster, Albert-Schweitzer-Campus 1, 48149 Münster, Germany; 14 LWL-University Hospital Hamm for Child and Adolescent Psychiatry, Ruhr-University Bochum, Heithofer Allee 64, 59071 Hamm, Germany

**Keywords:** Vitamin D [25(OH)D], C-reactive protein (CRP), Inflammation, Depression, Youth, Children and adolescents

## Abstract

**Purpose:**

Studies in adults suggest antidepressant effects of vitamin D, possibly via anti-inflammatory pathways, but evidence in youth is lacking. In the first randomized controlled trial (RCT) in depressed, vitamin D-deficient adolescents (DRKS00009758), symptom reduction emerged in only one of three outcomes. This secondary analysis investigates whether baseline inflammatory status modifies the effect of vitamin D.

**Methods:**

Data from 92 participants (78.3% ♀) of the RCT conducted at the Department of Child and Adolescent Psychiatry were analyzed. All participants were deficient in vitamin D [25(OH)D ≤ 30 nmol/l] and at least mild depressive symptoms [Beck Depression Inventory-II (BDI-II) ≥ 14]. Participants received 2640 IU/day of vitamin D or placebo for 28 days plus treatment as usual. Depressive symptoms were assessed via BDI-II and the Diagnostic System for Mental Disorders in Childhood and Adolescence (DISYPS), self- and proxy-rated. C-reactive protein (CRP) was measured at baseline as an inflammatory marker. Its impact on treatment effects was examined via interaction analyses and stratification by CRP status.

**Results:**

In 81.5% of the participants, CRP levels were below the threshold for low-grade inflammation (3 mg/L). No consistent interaction between treatment and baseline CRP was observed across outcomes. A nominal interaction for self-rated DISYPS at the 3 mg/L threshold lost significance after correction for multiple testing. Stratified analyses using different CRP thresholds revealed no significant subgroup effects.

**Conclusions:**

Overall, our findings do not support the hypothesis that the antidepressant effects of vitamin D supplementation in children and adolescents depend on baseline CRP status—at least within a sample characterized by minimal baseline inflammation.

## Introduction

Depression is a prevalent mental health disorder with a complex etiology [1] and one of the leading causes of disability worldwide [2]. In youth, depression is associated with impairments in academic performance, social interactions [3, 4], and quality of life [5], as well as increased morbidity and mortality risks [6, 7]. Notably, over 50% of all initial depression diagnoses are made before age 14, and 75% occur before age 24 [8], highlighting adolescence as a critical window for intervention. Vitamin D supplementation has shown promise as a treatment approach in meta-analyses of randomized controlled trials (RCTs) in adults [9, 10]. However, evidence in youth remains limited. The only RCT in children and adolescents conducted by our study group reported inconsistent results: only one of three depression measures showed significant symptom reduction [11]. While an inverse relationship has been observed in adults, the effects in youth were less clear, suggesting that vitamin D efficacy may depend on distinct biological subgroups and mechanisms.

There is increasing clinical and experimental evidence linking depression to inflammation [12, 13], with neuroinflammatory processes emerging as a key factor in its pathophysiology [14, 15]. Elevated levels of the inflammation marker C-reactive protein (CRP) have been reported in a subset of adults with depression [16], particularly in those with treatment-resistant symptoms [17]. Similar associations have been observed in children and adolescents [18]. Moreover, longitudinal studies associate elevated CRP levels in adolescence with the development of depressive episodes later in life, highlighting its potential as a biomarker for inflammatory pathways involved in the etiology of depression [19, 20]. While CRP ≥ 5 mg/L has been defined as the clinical threshold for acute inflammation in most medical conditions [21], low-grade inflammation in depression is defined as CRP ≥ 3 mg/L according to Centers for Disease Control and Prevention / American Heart Association (CDC/AHA) guidelines [22].

Considering the potential role of inflammation in the etiology of depression, anti-inflammatory agents, including vitamin D, could represent an option for the treatment of depression. Since previous studies indicated that vitamin D supplementation reduces CRP [23], vitamin D may exert antidepressant effects via immunomodulation. However, the proposed mechanisms of vitamin D in the context of depression are largely based on preclinical research [24]. While it has been emphasized that inflammatory markers should be evaluated alongside vitamin D in depression studies [9], longitudinal data on the interplay between vitamin D, inflammation, and depressive symptoms in children and adolescents are currently lacking. It remains unclear whether the antidepressant effects of vitamin D supplementation are more pronounced in subgroups with increased inflammation. Therefore, we conducted a secondary analysis of the RCT in adolescents [11] to examine whether baseline CRP levels modify the effect of vitamin D supplementation on depressive symptoms in this age group.

## Materials and methods

### Study design, participants and intervention

This secondary analysis is based on a randomized, double-blind, placebo-controlled trial conducted at the Department of Child and Adolescent Psychiatry, Psychosomatics and Psychotherapy, University Hospital Essen, Germany (LVR-Universitätsklinik Essen), between June 2016 and May 2018 [25]. The study was approved by the local Ethics Committee (No. 15–6363-BO), registered at the German Clinical Trials Register (DRKS00009758), and conducted in accordance with the Declaration of Helsinki. Eligible participants were in- or daycare patients aged 11.0–18.9 years with written informed consent who were at risk for vitamin D deficiency (serum 25(OH)D < 12 ng/mL [< 30 nmol/L]) [26] and had at least mild depressive symptoms (Beck Depression Inventory-II (BDI-II) score ≥ 14) [27]. Exclusion criteria were severe somatic or renal disease, intellectual disability (IQ < 70), established hypocalcemia, and/or parathyroid hormone level > 130 ng/mL. Screening for eligibility occurred within the first two days after admission [25]. While data from 100 participants were analyzed in the original RCT [11], only those with available CRP data (n = 92) were included in the present analysis.

Details of the intervention and randomization process are described elsewhere [25]. In short, all participants received treatment as usual (TAU) according to current clinical guidelines for their respective mental disorders [25]. Using computer-generated lists, participants were randomized (1:1) to receive either oral vitamin D₃ supplementation (2640 IU/day, i.e., 66 µg/day; verum group [VG]) or placebo (placebo group [PG]) for 28 days, starting no later than the fifth day after admission. Vitamin D₃ and placebo were provided as identical pills (Dr. B. Scheffler Nachf. GmbH & Co. KG, Bergisch Gladbach, Germany) in containers labeled with the randomization number. Participants were instructed to take three pills each morning after breakfast. Participants discharged from psychiatric treatment at the clinic at their own request before day 28 (n = 5) were instructed to continue supplementation. Furthermore, they received weekly telephone follow-ups and attended the end-of-study examination on day 28 post-randomization.

### Outcome and covariate assessments

The severity of depressive symptoms was assessed at baseline (T0) and post-intervention (T1) using the BDI-II (n = 92) [28], and the Diagnostic System for Mental Disorders in Childhood and Adolescence (DISYPS) based on self-reports (DISYPS Self; n = 92), and external assessment (DISYPS Proxy; n = 91) [29]. The BDI-II is a 21-item self-report questionnaire based on the Diagnostic and Statistical Manual of Mental Disorders, 4th Edition (DSM-IV) criteria, rated on a 0–3 Likert scale per item, with higher scores reflecting greater severity [28]. The DISYPS comprises 29 items (0–3 points each), referencing DSM-IV and the International Statistical Classification of Diseases and Related Health Problems Revision 10 (ICD-10) criteria [29]. It was conducted both as a self-assessment (DISYPS Self) and as an external assessment by guardians (DISYPS Proxy). Total scores were converted into stanine values, with scores ≥ 8 considered clinically relevant [29].

At T0 and T1, fasting blood samples were collected in the early morning from an antecubital vein into monovettes (Sarstedt, Nümbrecht, Germany), transferred to the Central Laboratory of the University Hospital Essen, and analyzed within one hour [25]. Serum 25(OH)D was measured using the Siemens ADVIA Centaur® Immunoassay-system (Siemens Healthineers, Erlangen, Germany). Serum CRP concentrations (mg/L) were determined at T0 using the Siemens Atellica® CH Analyzer (Siemens Healthineers, Erlangen, Germany). Clinical assessments at admission included assessments of body weight and height, pubertal status, and general health screening [25]. For participants under 18.25 years, the BMI standard deviation scores (SDS, kg/m2) were calculated using German national reference data according to the Lambda-Mu-Sigma (LMS) method [30].

### Statistical analysis

Sample characteristics at T0 and T1 were summarized by median (P25, P75) values for continuous variables, and percentages for categorical variables (Table 1). To address potential sex-specific differences, interaction analyses (treatment × sex assigned at birth) were conducted for each outcome (BDI-II, DISYPS Self, DISYPS Proxy) separately, with CRP, treatment, and sex assigned at birth as covariates in the model. As these analyses did not provide evidence of sex interactions, data from both sexes were pooled for further analyses. Intervention effects on depressive symptoms were analyzed separately for each outcome using analyses of covariance (ANCOVA) (Table 2). In the basic model, post-intervention values (T1) served as dependent variables, with treatment group (PG vs. VG) as the independent variable. Covariates were age, sex assigned at birth, baseline 25(OH)D concentration, and corresponding baseline values of the outcome, following the approach of Libuda et al. (2020) [11]. To examine the role of inflammation, baseline CRP concentration (T0) was added as an additional covariate (Model 1, Table 2). In subsequent interaction analyses, CRP was tested as a potential effect modifier via treatment × CRP interaction terms with CRP and treatment as covariates in the model. These analyses aimed to determine whether treatment effects on depressive symptoms significantly differed depending on baseline CRP levels. CRP was examined using three thresholds: (1) the clinically established threshold of 5 mg/L [21], (2) the 3 mg/L threshold proposed in recent research on depression [15, 31], and (3) the sample-specific median (0.845 mg/L). To account for multiple testing, *p*-values were corrected using the false discovery rate (FDR) method [32]. Afterwards, stratified analyses were conducted to assess treatment effects in CRP-defined subgroups using the thresholds defined above. All stratified models were adjusted for the covariates (Table 2). To assess the robustness of results, sensitivity analyses were conducted on four subsets: (1) participants not taking antidepressants [n = 78], due to potential effects on inflammation [33, 34], (2) participants without anorexia nervosa [n = 84], due to its impact on immunological processes [35, 36], (3) participants without potential current infection [n = 89], defined as CRP ≥ 10 mg/L [15], and (4) participants with clinically confirmed depression [n = 76], based on psychiatric assessment (*data not shown*). Analyses were performed using SAS 9.4 (SAS Institute Inc., Cary, USA). The alpha level was set to 0.05.

## Results

### Sample characteristics

A total of N = 92 participants aged 12.7–18.9 years were included in the analysis. Baseline characteristics (T0) were largely comparable between groups, but the PG had a higher proportion of females (82.2%) than the VG (74.5%) (Table 1). 15.2% were taking antidepressants (PG: 11.1%; VG: 19.1%). At T0, depressive symptomatology was pronounced in both groups, with median BDI-II scores of 28 and stanine scores of 9 on both DISYPS Self and Proxy—the highest possible score. From T0 to T1, median BDI-II scores improved slightly in both groups (VG: 28 to 25; PG: 28 to 23). While DISYPS Self and DISYPS Proxy scores remained stable in the PG, scores in the VG showed a slight improvement (Table 1). Baseline 25(OH)D levels were similarly low across groups, with median concentrations ≤ 9 ng/mL. After intervention (T1), the VG reached median levels in the “adequate” range (≥ 20 ng/mL). For CRP, baseline concentrations were comparable between groups (PG: 0.84 mg/L; VG: 0.85 mg/L; sample-specific median: 0.845 mg/L) (Table 1). Most participants had CRP levels below the clinical threshold of 5 mg/L (91.3%; n = 84) and below the 3 mg/L threshold for low-grade inflammation (81.5%; n = 75) (Table 2).

**Table 1:** Characteristics of N = 92 randomized participants in placebo (n = 45) or Vitamin D supplementation group (n = 47) at baseline (T0) and post-intervention (T1)

	Placebo group		Verum group	
	n	Median or %	n	Median or %
		T0		T0
Age [years]	45	16.1 (14.8; 17.4)	47	16.3 (15.2; 17.1)
Females [%]	37	82.2	35	74.5
Body height [cm]	45	164.7 (160.5; 169.4)	47	165.7 (162.0; 175.0)
Body weight [kg]	45	56.4 (50.4; 65.2)	47	65.0 (53.9; 75.0)
BMI-SDS [kg/m^2^]^a^	44	0.24 (−0.54; 1.49)	45	0.21 (−0.16; 1.79)
Antidepressants (Yes)	5	11.1	9	19.1
CRP [mg/L]	45	0.84 (0.32; 2.23)	47	0.85 (0.24; 2.19)

		T0	T1		T0	T1
25(OH)D [ng/mL]^b^	45	8.5 (6.8; 10.2)	9.4 (7.3; 11.5)	47	9.0 (7.7; 10.3)	24.7 (20.5; 27.6)
BDI-II (Total scores)	45	28 (23; 34)	23 (13; 36)	47	28 (22; 43)	25 (14; 42)
DISYPS Self (stanine)	45	9 (8; 9)	9 (8; 9)	47	9 (8; 9)	8 (8; 9)
DISYPS Proxy (stanine)	44	9 (8; 9)	9 (8; 9)	47	9 (8; 9)	8 (7; 9)

25(OH)D, 25-hydroxy-cholecalciferol; BDI-II, beck depression inventory II; BMI, body mass index; CRP; C-reactive protein; DISYPS Self, diagnostic system for mental disorders in childhood and adolescence based on self-assessment; DISYPS Proxy, DISYPS based on external assessment; n, number of participants

Values are presented as median (P25; P75) for interval-scaled variables, and percentages for categorical variables

^a^BMI-SDS calculated using German reference data (LMS method) for participants < 18.25 years [30]

^b^1 ng/mL serum 25(OH)D equates to 2.5 nmol/L

### Effect modification by baseline CRP levels

In the Basic model, a significant effect of vitamin D supplementation was observed only for DISYPS Proxy (Table 2). Adjusting for baseline CRP levels did not alter the results (Model 1, Table 2). No effect of baseline CRP was observed on either outcome. No significant interaction between treatment and CRP status at baseline was found when stratified by the threshold of 5 mg/L. In CRP-defined subgroups, no significant treatment effects were observed across all outcomes, including DISYPS Proxy. At the 3 mg/L threshold, a statistically significant interaction was found for DISYPS Self (*p* = 0.047), however, this did not remain significant after FDR correction (*p* = 0.423). Stratified analyses by baseline CRP level indicated opposing trends between groups, with a trend toward symptom improvement in the CRP ≥ 3 mg/L subgroup receiving verum, though no effects in CRP-defined subgroups reached significance. Analyses using the sample-specific CRP median (0.845 mg/L) revealed no significant interactions for any outcome. For DISYPS Self, the stratified results nevertheless followed a descriptively similar pattern to that observed at the 3 mg/L threshold. However, none of these subgroup effects reached significance (Table 2). These findings were consistent across sensitivity analyses (*data not shown*).

**Table 2:** ANCOVA^a^ results for Vitamin D intervention (verum) effects on depressive symptoms: interaction and stratified analyses by multiple CRP thresholds

Model	Predictor	BDI-II			DISYPS Self			DISYPS Proxy		
		n	β (CI)	*p*	n	β (CI)	*p*	n	β (CI)	*P*
Basic model^a^	verum	92	1.17 (−2.59; 4.93)	0.54	92	−0.14 (−0.69; 0.40)	0.59	91	−0.62 (−1.22; −0.03)	0.04
Model 1^b^	verum	92	1.46 (−2.35; 5.26)	0.45	92	−0.11 (−0.66; 0.45)	0.70	91	−0.61 (−1.21; −0.01)	0.047
	CRP		−0.29 (−0.89; 0.31)	0.34		−0.04 (−0.13; 0.04)	0.32		−0.02 (−0.11; 0.08)	0.32
*Basic model stratified by CRP ≥ 5.0 mg/L*^*c*^										
CRP ≥ 5.0 mg/L^a^	verum	8	1.90 (−13.84; 17.64)	0.73	8	−1.28 (−4.24; 1.68)	0.26	8	−0.45 (−3.37; 2.48)	0.66
CRP < 5.0 mg/L^a^	verum	84	1.24 (−2.79; 5.27)	0.54	84	−0.07 (−0.66; 0.53)	0.82	83	−0.64 (−1.29; 0.01)	0.05
		*p value for interaction*^e^ = 0.79			*p value for interaction*^e^ = 0.37			*p value for interaction*^e^ = 0.74		
*Basic model stratified by CRP ≥ 3.0 mg/L*^*d*^										
CRP ≥ 3.0 mg/L^a^	verum	17	2.34 (−2.34; 7.02)	0.30	17	−0.76 (−2.10; 0.58)	0.24	17	−1.25 (−3.36; 0.85)	0.22
CRP < 3.0 mg/L^a^	verum	75	1.73 (−2.57; 6.03)	0.42	75	0.10 (−0.49; 0.69)	0.73	74	−0.45 (−1.07; 0.18)	0.16
		*p value for interaction*^e^ = 0.26			*p value for interaction*^e^ = 0.047^f^			*p value for interaction*^e^ = 0.13		
*Basic model stratified by CRP median*										
CRP ≥ 0.845 mg/L^a^	verum	46	−0.01 (−5.29; 5.27)	0.99	46	−0.29 (−1.15; 0.56)	0.49	46	−0.78 (−1.56; 0.003)	0.05
CRP < 0.845 mg/L^a^	verum	46	3.88 (−2.18; 9.95)	0.20	46	0.23 (−0.50; 0.95)	0.53	45	−0.45 (−1.52; 0.61)	0.40
		*p value for interaction*^e^ = 0.34			*p value for interaction*^e^ = 0.53			*p value for interaction*^e^ = 0.70		

BDI-II, beck depression inventory II; CRP, C-reactive protein; DISYPS Self, diagnostic system for mental disorders in childhood and adolescence based on self-assessment; DISYPS Proxy, DISYPS based on external assessment; n, number of participants

^a^Basic model adjusted for age, sex assigned at birth, baseline 25(OH)D, and baseline value of the respective outcome

^b^Model 1: Basic model further adjusted for CRP

^c^Threshold of 5 mg/L, as clinically established [21]

^d^Threshold of 3 mg/L, as proposed in recent depression research [15, 31]

^e^*p value* for the interaction term (treatment × CRP) with CRP and treatment as covariates

^f^False discovery rate–adjusted *p *value = 0.423

## Discussion

To our knowledge, this is the first study to examine whether baseline inflammation modifies the effect of vitamin D supplementation on depressive symptoms in children and adolescents. Among 92 participants with vitamin D levels in the deficiency-risk range and at least mild depression, 81.5% had CRP levels below the threshold for low-grade inflammation (3 mg/L). No consistent interaction between CRP status and treatment effects emerged across outcomes and CRP cut-offs. An interaction for DISYPS Self at the 3 mg/L threshold was nominally significant but did not remain after FDR correction.

Depressive symptoms were assessed using three instruments, with only marginal reductions observed across all evaluation tools in both groups. These minimal changes may reflect the short intervention period, as previous studies suggest that four weeks may be insufficient to induce substantial neurobiological or antidepressant effects, with more improvements typically observed in longer trials [37]. However, adequate vitamin D levels (≥ 20 ng/mL) were reached in this short-time intervention in a population initially at risk for deficiency and DISYPS Proxy indicated a significant effect with adjunctive vitamin D treatment within this relatively short-term intervention. Thus, the study offers potential for preliminary insights into the relationship between vitamin D and depression.

Considering results from a previous meta-analysis [14], we defined low-grade inflammation as CRP ≥ 3 mg/L. Using this threshold, Osimo et al. (2019) reported elevated odds of low-grade inflammation in depressed patients (OR = 1.46). Furthermore, this meta-analysis showed that mildly elevated CRP ≥ 1 mg/L could be a relevant threshold in the etiology of depression as well [15]. Studies have shown elevated CRP levels in a subset of individuals with depression [15, 16, 18], particularly in those with treatment-resistant depression [17]. In contrast, despite only marginal reductions in depressive symptoms following the intervention, which included treatment as usual in both groups and suggests treatment resistance, we observed notably low inflammation levels in our cohort, and no effect of CRP on either outcome was observed. Only 18.5% exceeded CRP ≥ 3 mg/L, and 45.7% exceeded ≥ 1 mg/L, compared with the 27% and 58% reported by Osimo et al. in adults [15]. These lower rates in our adolescent cohort underscore that inflammation is not a universal feature of depression, particularly in youth, and suggest that alternative mechanisms may be more relevant in this age group. Further research is needed to clarify the role of inflammation across developmental stages and depressive subtypes [38]. The low overall CRP levels and limited variance in our sample may have reduced the ability to detect modifying effects. Furthermore, given that anti-inflammatory treatments appear effective mainly in patients with elevated inflammation [15], our sample with a predominantly subclinical inflammatory profile may have been inappropriate to elicit more obvious treatment effects of vitamin D. Future vitamin D interventions might focus on depressed individuals with CRP ≥ 3 mg/L, as it is already being done in current RCTs on pharmacotherapy [39].

Regarding the role of inflammation on the effect of vitamin D supplementation, adjusting for CRP did not alter the overall treatment effects, suggesting that inflammation, as measured by CRP, does not influence vitamin D efficacy in this cohort. Although different CRP cut-offs for inflammation have been proposed, including CRP > 5 mg/L [21], CRP ≥ 3 mg/L, and CRP ≥ 1 mg/L [15] (with the latter being close to our sample-specific median of 0.845 mg/L), none of these thresholds revealed interactions with treatment effects. Additionally, an interaction test with CRP as a continuous variable also showed no significant results, reinforcing that baseline inflammation does not appear to modify the effect of vitamin D supplementation. Stratified analyses showed a slight reduction in depressive symptoms in participants with CRP ≥ 3 mg/L in the DISYPS Self (Δ = −0.76), while those of participants with lower CRP remained unchanged. A similar pattern was observed at the median CRP threshold. While these findings may support the hypothesis that individuals with higher CRP levels benefit more from vitamin D supplementation, it must be emphasized that none of the subgroup effects reached significance. These findings are consistent with those of Kaviani et al. (2022), who observed BDI-II reductions after 8 weeks of high-dose vitamin D supplementation in adults despite lacking changes in inflammatory markers [40]. Similar to our cohort, baseline CRP levels in this study were low, indicating no elevated inflammatory status prior to the intervention. Although they did not formally test interaction effects, their results suggest that the antidepressant effects of vitamin D may occur independently of inflammation [40]. Likewise, Dogan-Sander et al. (2021) examined the relationship between inflammation and the effect of vitamin D on depressive symptomatology in a cohort of adults, with similarly low baseline CRP levels. Despite observing a correlation between low vitamin D levels and increased depressive symptoms, no moderating effect of inflammation was found [41], supporting our suggestion that vitamin D effects on depression may be independent of baseline inflammation.

Overall, our results suggest that CRP as a proxy for inflammation, plays a limited role in modulating vitamin D’s antidepressant effects in the presented cohort. This challenges the hypothesis that anti-inflammatory effects explain vitamin D effect on depression, at least in populations with low grade inflammation. While the question of defining the optimal CRP threshold remains unresolved, the absence of significant interactions suggests that other mechanisms of vitamin D may be more relevant in adolescents with non-elevated inflammation. These could include the antioxidative and neuromodulatory properties of vitamin D, as well as its role in serotonin synthesis and receptor modulation, as highlighted in previous mainly preclinical studies [24]. However, it remains unclear to what extent these mechanisms translate to clinical effects in children and adolescents, and whether mechanisms of action differ from those observed in adults [24].

### Strength and limitations

Recruitment from a clinical setting enhances internal validity, though generalizability is limited, as only about 20% of children with mental disorders receive psychiatric care [42–44]. However, this clinical setting allows for a more homogenous and well-characterized sample. All participants met the criteria for at least mild depression [27, 29], and baseline vitamin D levels indicated deficiency risk [26]. Information on acute infections or inflammatory conditions at the time of assessment was not systematically collected. However, following Osimo et al. (2019), CRP ≥ 10 mg/L was used as an indicator of potential current infection [15]. Only n = 3 participants exceeded this threshold, and a sensitivity analysis excluding these cases did not alter the study results (*data not shown*).

Depressive symptoms were assessed using three instruments, including self- and proxy-rated ratings [28, 29]. Discrepancies between self- and proxy-reports are common in child and adolescent psychiatry [45–47], as parents often emphasize symptom severity while adolescents tend to under-report symptoms [48]. This may lead to inconsistencies and potential misclassification. In our study, only parent-reported (DISYPS Proxy) symptoms showed significant effects in the unstratified analyses, whereas a nominal effect emerged for self-reported DISYPS in the interaction analyses, but not for the BDI-II. To account for such discrepancies, we followed best practices in youth mental health through a multi-informant approach [45, 46]. As the applied instruments assess symptom severity rather than diagnostic status [28, 29], depression was additionally verified by clinical evaluation, which classified 76 of 92 participants as depressed. A sensitivity analysis restricted to these 76 participants did not change the results (*data not shown*).

Focusing solely on CRP limits interpretation, as it reflects only part of the immune response. Additional humoral and cellular markers, such as cytokines (e.g., TNF-α, IFN-γ, IL-1β, IL-6, IL-8, IL-10) and immune cell subsets, as proposed in other studies exploring the etiology of depression [16, 18, 49–53], may capture distinct pathways that CRP alone cannot represent. These markers could provide a more nuanced understanding of the immune mechanisms underlying adolescent depression and how inflammation influences depressive symptoms and treatment response. While additional markers could enhance the analysis, CRP remains a valid and well-supported choice for investigating inflammation in depression, with associations to depression severity [16, 18], and well-established cut-offs [15, 31]. CRP was only analyzed at baseline, limiting insights into temporal dynamics. While this was sufficient for examining the potential modulating effect of inflammation on vitamin D supplementation, repeated assessments could provide a deeper understanding of how inflammation develops over time following vitamin D treatment. Given evidence that depression and inflammation may influence each other during childhood and adolescence [18], future studies incorporating multiple time points could clarify how these dynamics impact the long-term effects of vitamin D supplementation on depression.

While sex-specific patterns in the inflammation–depression link have been suggested [54], other studies report no association between CRP levels and sex or gender [15]. In our study, no interaction between sex and treatment was observed. Yet, the overall sample size was too limited to allow for meaningful sex-stratified analyses. Such stratification would have further reduced statistical power and reliability. Nonetheless, we adjusted for sex in all ANCOVA models. The relatively small sample size, particularly in inflammation-defined subgroups (e.g., n = 8 for CRP ≥ 5 mg/L), limited statistical power and may have obscured subtle effects. While this constraint must be acknowledged, it reflects the practical challenges of recruiting clinically well-characterized adolescents for biological studies. Nonetheless, the present findings provide a valuable foundation for future trials, highlighting key methodological aspects—such as adequate stratification by inflammatory status and longer intervention durations—that can guide more targeted study designs.

## Conclusions

Overall, our findings do not support the hypothesis that the antidepressant effect of vitamin D supplementation in children and adolescents depends on CRP status—at least within a sample characterized by non-elevated inflammatory levels. Larger vitamin D RCTs with longer intervention periods are needed to investigate the role of more pronounced inflammation, ideally assessed using a broader range of inflammatory markers.

## Data Availability

No data was used for the research described in the article.
